# Preparedness of HIV care and treatment clinics for the management of concomitant non–communicable diseases: a cross–sectional survey

**DOI:** 10.1186/s12889-016-3661-1

**Published:** 2016-09-21

**Authors:** Claudia Leung, Eric Aris, Aisa Mhalu, Hellen Siril, Beatrice Christian, Happiness Koda, Talumba Samatta, Martha Tsere Maghimbi, Lisa R. Hirschhorn, Guerino Chalamilla, Claudia Hawkins

**Affiliations:** 1 Feinberg School of Medicine, Northwestern University, 420 E Superior Street, Chicago, IL 60611 USA; 2 Management and Development for Health HIV/AIDS Care and Treatment Program, P.O. Box 79810. Plot #802, Mwai Kibaki Road, Mikocheni, Dar es Salaam, Tanzania; 3 Department of Global Health and Social Medicine, Harvard Medical School, 401 Park Drive 3East, Boston, MA 02215 USA

**Keywords:** HIV, Non–communicable diseases, Tanzania, Health system strengthening, Integration

## Abstract

**Background:**

In Sub-Saharan Africa, epidemiological studies have reported an increasing burden of non-communicable diseases (NCD) among people living with HIV. NCD management can be feasibly integrated into HIV care; however, clinic readiness to provide NCD services in these settings should first be assessed and gaps in care identified.

**Methods:**

A cross-sectional survey conducted in July 2013 assessed the resources available for NCD care at 14 HIV clinics in Dar es Salaam, Tanzania. Survey items related to staff training, protocols, and resources for cardiovascular disease risk factor screening, management, and patient education.

**Results:**

43 % of clinics reported treating patients with hypertension; however, only 21 % had a protocol for NCD management. ECHO International Health standards for essential clinical equipment were used to measure clinic readiness; 36 % met the standard for blood pressure cuffs, 14 % for glucometers. Available laboratory tests for NCD included blood glucose (88 %), urine dipsticks (78 %), and lipid panel (57 %). 21 % had a healthcare worker with NCD training. All facilities provided some form of patient education, but only 14 % included diabetes, 57 % tobacco cessation, and 64 % weight management.

**Conclusions:**

A number of gaps were identified in this sample of HIV clinics that currently limit the ability of Tanzanian healthcare workers to diagnose and manage NCD in the context of HIV care. Integrated NCD and HIV care may be successfully achieved in these settings with basic measures incorporated into existing infrastructures at minimal added expense, i.e., improving access to basic functioning equipment, introducing standardized treatment guidelines, and improving healthcare worker education.

## Background

The burden of non–communicable diseases (NCD) is increasing in Sub–Saharan Africa (SSA). Among people living with HIV (PLWH), epidemiological studies reflect a trend of increasing prevalence and incidence of four major risk factors of cardiovascular disease, hypertension, hyperglycemia, dyslipidemia, and obesity [1–7]. Behavioral risk factors for NCD (i.e., smoking, alcohol use) are more common among PLWH [8–10], and with the increasing availability of antiretroviral therapy (ART), the ageing–HIV population is also susceptible to traditional risk factors for NCD. Furthermore, both the HIV virus and prolonged ART use have been associated with dyslipidemia, insulin resistance, and atherosclerosis, interacting with traditional risk factors of NCD [11–15]. The increased NCD risk among PLWH has the potential to threaten the success of ART, causing premature morbidity and mortality. It is therefore crucial to identify and manage NCD-related risk factors in this patient population.

SSA the only region in the world where infectious disease deaths still outnumber those of NCD [16]. Consequently, both external funding as well as healthcare services focus predominantly on the treatment of communicable diseases [17] and resources are significantly lacking for the prevention and management of NCD [18, 19]. Both HIV and NCD are conditions that require a continuum of care, frequent laboratory and clinical monitoring, behavioral changes and adherence support. Many tools and systems in place for HIV care can be adapted for the prevention and management of some of the leading NCD. Successful integration of HIV and NCD care has already been demonstrated in a few SSA countries. In Ethiopia, findings from a pilot study conducted in diabetes clinics demonstrated significant improvements in standards of care by utilizing strategies developed for HIV care for NCD, including step-by-step protocols, family-focused care, and identification of simple but useful monitoring and evaluation indicators [20]. Supported by initiatives by the World Health Organization (WHO) and the Gates Foundation, providing NCD care to PLWH may not require a new vertical platform of care, but rather integrated health services that can draw lessons and leverage from existing models of care [21, 22]. To achieve this, existing HIV clinics need adequately trained staff, equipment, medications, and protocols for NCD identification and management.

The objective of this study was to describe the facility resources available for NCD diagnosis and treatment in U.S. President’s Emergency Plan for AIDS Relief (PEPFAR)–supported HIV Care and Treatment Clinics (CTCs) in Dar es Salaam, Tanzania, and identify areas where existing services need to be strengthened to more effectively incorporate NCD prevention and management with HIV care.

## Methods

### Study setting

This cross–sectional survey was conducted in July 2013 at 14 Management and Development for Health (MDH)–supported HIV CTCs in the three districts of Dar es Salaam, Tanzania. The facilities were chosen by convenience sampling within district and facility size strata: eight large health centers, and six small dispensaries. All were public facilities with functioning CTCs at the time of assessment. The CTCs are outpatient facilities that range in size from small dispensaries to large hospital–affiliated clinics, and while some are adjacent to hospitals or general practice clinics, they remain separate in staff, operation, and facilities. MDH provides technical support with PEPFAR funds to 49 private and 50 public HIV CTCs including district and tertiary referral hospitals, health centers and dispensaries. Dar es Salaam has an estimated HIV prevalence of 6.9 % among females and 5.3 % among males [23]. At the time of this study, 129,892 HIV-infected patients were active in care (male:female ratio 1.05). PLWH receive HIV care and ART free of charge and are seen monthly, receiving routine physical and laboratory testing including tuberculosis screening, Hepatitis B serology, CD4 count, HIV RNA quantification, comprehensive chemistries, blood glucose, and creatinine. Some non-ART including antibiotics and antimalarial treatments are also dispensed free of charge. Patients with NCD complications are referred to separate outpatient facilities for further management. Each CTC is staffed with physicians (medical officers or mid–level providers), nurses, phlebotomists, pharmacists or pharmacy technicians, and patient trackers.

### Survey

A 30 question survey assessing resources and services available for NCD diagnosis and management was administered in English to the HIV CTC Site Manager (medical officer or mid–level provider) or Site Supervisor (nurse) at each facility. The survey was adapted from a facility survey developed by Partners in Health to assess site readiness for ART initiation in HIV care facilities, which also included questions on resources available for NCD care [24].

Survey items were related to 1.) number and level of training of the staff in NCD, 2.) type and availability of services related to NCD, 3.) availability of equipment for NCD risk factor assessment, and 4.) NCD resources for patient education. “Staff with NCD training” was defined as any medical staff (physicians or nurses) that had received any training specific to the management of NCD beyond their standard medical training. “Treatment of NCD” was defined by the physician’s comfort to assess and manage risk factors for NCD and provide non-emergent NCD management. Equipment was considered “functional” if available and functioning at the time of survey, reflecting the ability to use that equipment in a patient encounter at that point in time. Examples of equipment considered “non-functional” were glucometers without strips and broken weighing scales.

### Analysis

The key study outcomes were the availability of services and resources needed for NCD care at the patient, provider and clinic level with a focus on the most common NCD and NCD risk factors, including hyperlipidemia, diabetes, and hypertension. Descriptive statistics were used to measure the frequency of service and resource availability for NCD diagnosis and management across the clinics.

## Results

### Facility characteristics and basic services

The 14 HIV CTCs participating in the survey ranged in size from small dispensaries to large hospital-affiliated clinics (referred to subsequently as “dispensaries” and “hospital clinics”). The total number of patients seen ranged from 43 to 446 patients per week (Table 1). The mean number of total patient visits per week was 69 for dispensaries and 330 for hospital clinics. The patient to healthcare provider (medical officers, mid-level providers, and nurses) ratio was 23.1 for dispensaries and 26.2 for hospital clinics. All clinics provided pediatric and adult HIV care, point of care HIV testing, first line ART, tuberculosis screening, and family planning services.

**Table 1:** Characteristics of HIV CTCs per MDH quarterly report

Clinic	New Patients Seen/week	New Patients Started on ART/week	Total Patients Seen/week	Patient/Staff Ratio
Disp–1	1.4	1.2	42.8	2.9
Disp–2	4.8	2.9	44.8	22.4
Disp–3	2.5	1.8	54.3	27.2
Disp–4	3.7	2.5	68.6	17.2
Disp–5	3.6	2.4	96.2	48.1
Disp–6	7.9	5.2	105.6	21.1
Hosp–1	14.9	11.9	177.5	35.5
Hosp–2	17.2	15.2	243.4	20.3
Hosp–3	18.7	13.1	248.9	27.7
Hosp–4	22.1	23.1	314.4	31.4
Hosp–5	6.8	3.5	392.6	23.1
Hosp–6	14.2	15.1	435.8	20.8
Hosp–7	26.1	25.0	445.6	29.7
Hosp–8	20.8	18.1	445.9	21.2

Dispensaries (Disp) ≤ 150 total patients seen per week; Hospital Clinics (Hosp) >150 total patients seen per week

### Resources for the diagnosis and management of NCD

**Fig. 1:**
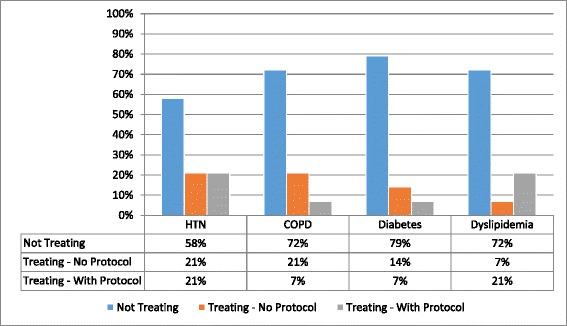
Percentage of facilities that treat NCD with or without protocol. The X-axis represents a selection of the NCDs included in the survey, with the corresponding percentages for diseases not treated, and treated with and without protocol

All 14 clinics exclusively treat patients with HIV. 43 % of clinics report treating patients with hypertension, however, only 21 % of facilities had a protocol for hypertension management available. 21 % and 29 % of the clinics treated patients with diabetes and dyslipidemia, respectively, but only 7 % and 21 % had a protocol for diabetes and dyslipidemia management respectively (Fig. 1. Percentage of facilities that treat NCD with or without protocol).

Treatment of NCD, specifically hypertension, chronic obstructive pulmonary disease (COPD), diabetes, and dyslipidemia was variable between dispensaries and hospital clinics. In dispensaries, 67 % treated patients with hypertension, 50 % with COPD, 16.7 % with diabetes, and 33.3 % with dyslipidemia. In comparison, 25 % of hospital clinics treated patients with hypertension, 12.5 % with COPD, 25 % with diabetes, and 25 % with dyslipidemia.

**Fig. 2:**
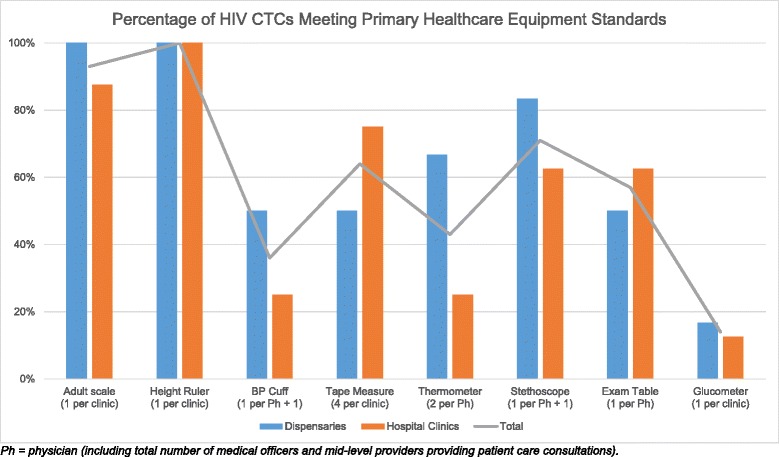
Percentage of HIV CTCs meeting primary health care equipment standards. The suggested standards for essential equipment in a primary health clinic according to the guidelines published by the ECHO International Health Resources are captured in the X-axis; the Y-axis refers to the percentage of facilities surveyed that meet these standards (dispensaries and hospital clinics separately, and then in total)

**Fig. 3:**
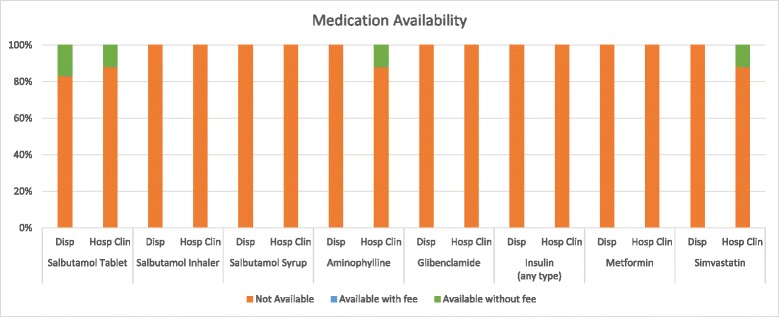
Medication availability. The X-axis represents the medications for NCD management included in the survey. The Y-axis represents the percentage of clinics with the corresponding medication availability

The ECHO International Health Resources guidelines are standards endorsed by the WHO for essential clinical equipment for primary healthcare clinics in low- and middle-income countries (LMIC) [25]. 71 % of surveyed facilities met the suggested standards for number of available stethoscopes. Other supplies found in over 50 % of facilities included height rulers (100 %), adult scales (93 %), tape measurers (64 %), and exam tables (57 %). However, only 36 % met the standard for blood pressure cuffs, and 14 % for glucometers (Fig. 2. Percentage of HIV CTCs meeting primary healthcare equipment standards). Dispensaries averaged meeting standards for 64.6 % of clinical equipment, while hospital clinics averaged meeting standards for 56.3 % of clinical equipment. Only two of 14 clinics (14 %) reported having any medications available in their pharmacy for the management of NCD (salbutamol tablets, aminophylline, and simvastatin), and all were dispensed free of charge (Fig. 3. Medication Availability).

93–100 % of clinics provided laboratory services for the diagnosis of infectious diseases, including HIV, malaria, syphilis, tuberculosis, and hepatitis B and C. Renal and liver function testing were also widely available. Tests for NCD-related conditions were lower including 88 % with blood glucose testing available, 78 % urine dipsticks, and 57 % lipid panel.

### Healthcare worker and patient education

21 % of facilities employed a healthcare worker (HCW) with NCD training, approximately 4.3 % of total facility HCWs. None of the facilities employed a specialist in NCD (i.e., cardiologist).

**Fig. 4:**
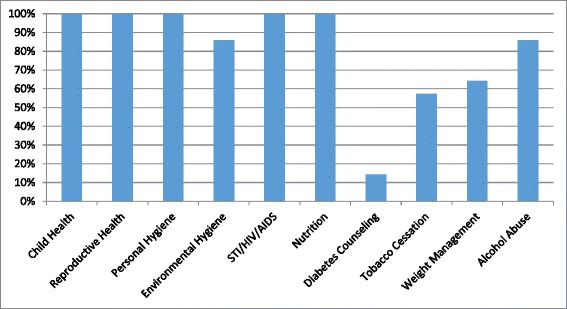
Health education topics availability. The topics for health education at the HIV CTCs are represented on the X-axis; the Y-axis refers to the percentage of facilities that teach on each topic

Patient education was provided in all of the surveyed CTCs, and 57 % of clinics provided more than ten sessions weekly. While all clinics had health education topics including HIV/AIDS, child health, reproductive health, personal hygiene, and nutrition, only 14 % included diabetes, with higher rates of tobacco cessation (57 %), weight management (64 %), and alcohol abuse (86 %) (Fig. 4. Percentage of facilities offering select topics in patient education). All dispensaries covered at least three of five key NCD topics (Nutrition, Diabetes, Tobacco Cessation, Weight Management, Alcohol Abuse), while only 50 % of large clinics met this same benchmark. Additionally, patient education materials (i.e., posters, brochures, videos) were available in all facilities, but only 21 % of facilities had patient education materials related to NCD.

**Fig. 5:**
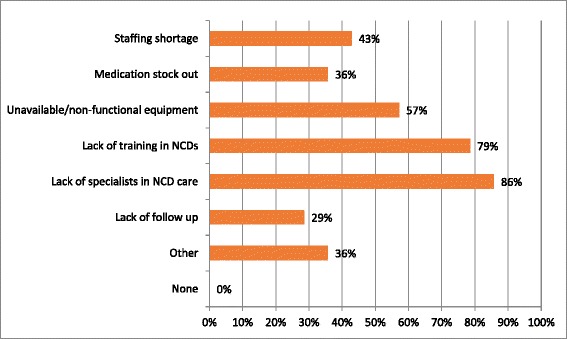
Perceived barriers to NCD care. Respondents were asked to report on their perceptions of patient barriers to NCD care in the HIV CTCs. Respondents were able to list as many barriers as applicable. None of the respondents reported no barriers to NCD care

All 14 respondents reported at least one gap in NCD care. More than 50 % reported a lack of equipment, HCW training in NCD, and access to specialist in NCD care (Fig. 5. Perceived barriers to NCD care as reported by the site manager).

## Discussion

In this survey of 14 HIV CTCs in Dar es Salaam, Tanzania, significant gaps in the services and resources available to diagnose and treat NCD were identified. These included a lack of access to treatment protocols, HCW training, and functioning equipment to adequately diagnose and manage NCD. This study is one of the first to report on the current capacity of HIV care and treatment facilities in SSA to screen and manage NCD.

While 50 % of the clinics reported treating NCD, many of these clinics reported managing NCD without access to protocols or training for disease management. Only 21 % of clinics had at least one healthcare worker trained in NCD. Farzadfar and colleagues demonstrated in rural and urban Iran that trained HCW and well-established guidelines for disease management are important factors for NCD prevention and management [26]. A clinic in Cambodia demonstrated over a 3-year period that HCW training for the management of lifelong diseases was effective for both HIV and diabetes care [27]. Importantly, guidelines are useful for standardization of medical care and optimize the utility of basic equipment, laboratory tests, and medications, particularly in settings where care is delivered primarily by mid-level providers and nurses. Guidelines also provide a means to decide which NCD-related complications are appropriate to be managed in HIV clinics, and when patients should be referred to a higher-level institution.

Overall, none of the surveyed facilities met all the ECHO International Health standards for essential clinic equipment used routinely in the diagnosis and management of NCD. Most clinics were equipped with functioning scales, however, only one-third had functioning blood pressure cuffs. Our results were similar to a program assessment of diabetes clinics in Swaziland in which variable access to basic equipment and NCD drugs was observed [20]. Similar gaps in access to basic technologies and essential medicines for NCD was found in a large multicenter WHO feasibility study of primary care facilities in eight LMICs [28]. While the ECHO standards for essential equipment are designed for a primary health clinic (rather than an HIV clinic) [25], used in the context of this survey, they provide a measurement of the readiness of the surveyed facilities for NCD care. Without reliable equipment, HCW will be unable to monitor and screen for important risk factors for NCD such as hypertension, nutritional status, and hyperglycemia. These represent missed opportunities within these clinics to provide reliable and adequate NCD care and save lives.

The majority of clinics did not stock medications for the management of NCD and NCD risk factors. Many of these medications are available through the outpatient pharmacy located in the general health clinics and hospitals often located adjacent to the HIV clinics (personal communication, A. Mhalu). However, their personnel and operations are largely separate, so it cannot be assumed that HIV patients have the same level of access to NCD care as those in the general health clinics. Encouragingly, basic lab testing including blood glucose, chemistry and lipid panel testing were found to be widely available, which is in contrast to surveys conducted in LMIC primary healthcare or other non-HIV clinics [20]. However, laboratory testing has the greatest utility if used in in a clinic that uses other comprehensive strategies for NCD screening and prevention. Ensuring access and availability of medications and laboratory tests for diagnosis and monitoring therefore is essential for NCD care.

We observed differences between small dispensaries and large hospital clinics with regard to NCD service provision and equipment availability, although due to the limited sample size, calculations for statistical significance were not performed. However, we noted that dispensaries tended to outperform their larger counterparts in terms of diseases treated (67 % vs. 25 % hypertension, 50 % vs. 12.5 % COPD, 33.3 % vs. 25 % dyslipidemia), equipment availability (64 % vs. 56.3 %), and education topics covered (100 % vs. 50 %), despite similar patient–provider ratios. This may be a reflection of the physicians in the smaller clinics electing to “do more” for their patients because smaller clinics are usually located further away from referral and specialist facilities.

An encouraging finding from the survey was the strong emphasis on patient education within the sites. All facilities offered weekly patient education sessions and individualized patient counseling during every patient visit. While the majority of sessions were devoted to maternal and child health and communicable diseases, these sessions offer a unique opportunity to educate patients on an array of NCD at no additional cost or personnel. A second strength was the high level of awareness of NCD in the clinic facilities. All of the survey respondents reported at least one gap in NCD care with a lack of training and equipment reported as the most significant limitations.

The study findings have significant implications for local Tanzanian policy. The gaps in our findings in NCD care can be grossly categorized in Paul Farmer’s four S’s, “stuff, staff, space, and systems” for effective care delivery [29]. HIV care in this setting is a framework – a “system” and a “space.” However, basic equipment, medications, staff training, and guidelines for NCD management are crucial to extending this system to include the identification and management of co-morbid NCD risk factors and to prevent the development of preventable, late-stage NCD complications (i.e., renal disease, myocardial infarction). Without a progressive approach towards medical care for this patient population, NCD threaten to undermine the success of ART for PLWH, causing premature morbidity and mortality.

Peck et al. recently reported on the preparedness of outpatient primary care facilities in Tanzania to manage NCD [4]. They report low rates of NCD care in general practice health centers and dispensaries, likely due to a lack of clear guidelines, basic supplies, appropriate medications, HCW training, and diagnostic equipment. Our study found similar gaps in NCD care in the HIV CTCs. This is in stark contrast to the strength of the HIV care model in Tanzania, which includes strong leadership by the Ministries of Health, national guidelines, HCW training opportunities, and available medication and diagnostic equipment.

Vertical, health-condition specific systems of care can create fragmented care that fosters inefficient resource utilization and gaps in care for patients with multi-morbidities [21]. While expanding NCD resources in general health clinics should be an eventual goal, leveraging the existing strengths of the HIV care system for NCD care may increase both the quality and efficiency of care delivery with minimal cost. Our study supports and expands upon the observations of Peck et al by identifying gaps in NCD care in existing HIV clinics, and supporting the authors’ recommendation that NCD care should be integrated with the complex primary care of HIV clinics [4]. Similarly, the WHO global strategy for People–Centered and Integrated Health Services calls for a fundamental shift in health care delivery away from vertical care systems towards integrated health care delivery to meet the burden of treating long-term, chronic conditions including HIV and NCD [21]. The implications of this study align with the Tanzania HIV and AIDS Strategic Plan, which includes a priority to implement HIV collaborative activities to reduce co-morbidities including cancers, hypertension, diabetes, and coronary heart disease [30]. In a resource-limited setting, the results of our survey provide a clearer understanding of the gaps in NCD care in outpatient HIV clinic settings that will help prioritize the most effective strategies for integrating NCD diagnosis and management into HIV care.

This study is limited by its sample population and type of data. The participating clinics in this survey were intended to be a representative sample of the public HIV CTCs in Dar es Salaam, the gold standard urban setting in Tanzania. CTCs included may not be representative of HIV care in the rest of the country, particularly more rural areas. However, it is likely that changes in HIV care in Dar es Salaam will have a ripple effect on other areas of Tanzania and SSA countries. Additionally, this study is based on self–reported data, and descriptive and cross–sectional in design, intending only to elucidate a qualitative understanding of the state of NCD care in HIV clinics, rather than report on outcomes. Additional studies examining quality of NCD care provided, evaluating effective and efficient care strategies at individual and health systems levels, and evaluating cost–effective prevention strategies are needed.

## Conclusions

An increasing burden of NCD among PLWH has been reported in Dar es Salaam, Tanzania, as well as other LMICs, and an integrated approach to NCD and HIV care is necessary to address NCD co–morbidities. Integration of NCD and HIV care should have three major targets: HCW training, access to comprehensive guidelines for NCD management, and improved access to basic functioning equipment, laboratory tests, and medications, many of which could be implemented at little additional expense. Patient education, the proximal location of HIV CTCs to general health clinics, and the expressed desire of HCWs to improve NCD care are strengths in the current system and should be leveraged.

## Abbreviations

ART: Anti-retroviral therapy
CTCs: Care and treatment clinics
HCW: Healthcare worker
HIV: Human immunodeficiency virus
LMIC: Low- and middle-income countries
MDH: Management and development for health
NCD: Non-communicable disease
PEPFAR: U.S. President’s Emergency Plan for AIDS Relief
PLWH: People living with HIV
SSA: Sub-Saharan Africa
WHO: World Health Organization
